# Serum Levels of Ficolin-3 and Mannose-Binding Lectin in Patients with Leprosy and Their Family Contacts in a Hyperendemic Region in Northeastern Brazil

**DOI:** 10.3390/tropicalmed7050071

**Published:** 2022-05-10

**Authors:** Francisca Jacinta Feitoza de Oliveira, Maria Aparecida Alves de Oliveira Serra, Leonardo Hunaldo dos Santos, Márcio Flávio Moura de Araújo, Rosemeire Navickas Constantino da Silva, Anete Sevciovic Grumach

**Affiliations:** 1 Social Sciences, Health and Technology Institute, Universidade Federal do Maranhão, Av. da Universidade, s/n Dom Afonso Felipe Gregory, Imperatriz 65915-240, Brazil; 2 Clinical Immunology, Centro Universitario FMABC, Santo André 09060-870, Brazil; rosemeire.constantino@fmabc.com (R.N.C.d.S.); anete@grumach.com (A.S.G.); 3 Programa de Pós-Graduação em Saúde e Tecnologia, Centro de Ciências Sociais, Saúde e Tecnologia, Universidade Federal do Maranhão, Eusébio 61773-270, Brazil; maa.oliveira@ufma.br (M.A.A.d.O.S.); leonardo.hunaldo@ufma.br (L.H.d.S.); 4 Fundação Oswaldo Cruz (FIOCRUZ), Euzébio 61760-000, Brazil; marcio.moura@fiocruz.br

**Keywords:** leprosy, infectious diseases, tropical disease, neglected disease, complement pathway, mannose-binding lectin

## Abstract

The present study aimed at analyzing the serum levels of mannose-binding lectin (MBL) and ficolin-3 (FCN3) in leprosy patients and their healthy family contacts in a hyperendemic region in northeastern Brazil. A cross-sectional study was carried out with 90 patients who had been diagnosed with leprosy and 79 healthy family contacts. Serum levels of the MBL and FCN3 proteins were measured using the immunofluorometric assay (ELISA). Clinical information was determined from the patients’ charts. It was observed that the leprosy patients were more likely to be male (OR = 2.17; *p* = 0.01) and younger than fifteen years of age (OR = 2.01; *p* = 0.03) when compared to the family contacts. Those under 15 years of age had higher levels of MBL (4455 ng/mL) than those over 15 years of age (2342 ng/mL; *p* = 0.018). Higher FCN3 levels were identified in patients with indeterminate leprosy (41.9 µg/mL) compared to those with the lepromatous form (34.3 µg/mL; *p* = 0.033) and in those with no physical disabilities (38.1 µg/mL) compared to those with some disability (*p* = 0.031). Higher FCN3 levels were also observed in the group of patients without leprosy reactions (37.4 µg/mL) compared to those with type 1 (33.7 µg/mL) and type 2 (36.1 µg/mL) reactions. The MBL levels were higher in children under 15 years of age than they were in adults. It was evidenced that higher FCN3 serum levels were associated with early and transient clinical forms and lower expression in severe forms of leprosy.

## 1. Introduction

Leprosy is an infectious disease that is caused by the intracellular bacillus *Mycobacterium leprae*, whose magnitude and disabling power make the disease a worldwide public health problem [1]. Although some regions of the world have achieved the reduction and elimination of the disease, countries such as India, Brazil, and Indonesia were responsible for 74% of the 127,396 new cases of leprosy registered in 2020 [2].

Brazil has the second highest number of new cases globally and the highest number in the Americas [2]. In particular, in northeastern Brazil, the state of Maranhão has the largest indexes of the disease, reaching detection rates of 44.94/per 100 thousand inhabitants [3,4]. The city of Imperatriz, the second largest of the state, presented high and very high levels of endemicity in 2008 and 2017, respectively, with prevalence rates ranging between 15.6 and 7.8/10 thousand inhabitants, making the fight against leprosy of prime importance [5].

The mechanisms by which *M. leprae* produces different clinical conditions in an infected person are not yet fully understood. Bacterial, individual, and environmental factors can influence the appearance of different disease manifestations. Among the main pathogenic mechanisms that are involved are the virulence factors of the bacteria, such as their long incubation time, and the immune response of the infected person [6,7].

The clinical manifestations of leprosy vary according to the host’s immune response and can be classified into two categories—lepromatous and tuberculoid—although many patients are in less defined intermediate groups, with leprosy taking on indeterminate and borderline forms [8]. Lepromatous leprosy is characterized by a low cellular immune response, a type 2 cytokine profile, a high bacillary load, and disseminated lesions and represents the most severe form of the disease. Tuberculoid leprosy has strong cell-mediated immunity, a type 1 cytokine profile, low bacillary load, and localized lesions. Indeterminate leprosy refers to an early stage of the disease that can progress to spontaneous healing or can develop into one of the other five forms [9].

Phagocyte activation and the T cell response are considered determinants for the progression and evolution of leprosy [10]. The complement system acts on the inflammatory response, opsonization, microbicidal activity, and interaction with adaptive immunity [6,7,8]. In the lectin pathway, for example, pattern recognition in sugars or acetylated residues by mannose-binding lectin (MBL), 11 collectin (CL-11), or by one of the three types of ficolins (FCN1, FCN2, and FCN3) induces a cascade of reactions that culminate in the phosphorylation, phagocytosis, or lysis of the target microorganisms through the formation of a membrane attack complex [11,12,13].

Lectins are molecules of great importance for the recognition and elimination of pathogens during the first and subsequent stages of immune defense. Few studies have evaluated the influence of the complement system in leprosy patients, and the current results are not yet well established. High levels of MBL appear to increase susceptibility to lepromatous leprosy in adult patients [14,15]. In contrast, low levels of MBL and disturbances in complement system activation can confer resistance against leprosy and borderline leprosy [16].

Studies have shown that polymorphisms in the FCN1 [17] and FCN2 [18] genes seem to protect people against serious illness. In comparison, another study did not observe an association between the polymorphism of the FCN3 gene with leprosy, although a high concentration of FCN3 has been found in patients with the lepromatous form of the disease [19].

The molecular mechanisms of *M. leprae* infection and immune evasion are still poorly understood [19]. It is believed that family contacts are the main risk group for developing leprosy due to greater exposure to the bacillus through prolonged conviviality. Therefore, following up with family contacts is a good strategy for early diagnosis and for monitoring disease transmission [20].

Therefore, investigating the immune response of leprosy patients and their family contacts will contribute to a better understanding of the different manifestations of the disease and will have implications for diagnosis and treatment in addition to disease prevention and early diagnosis in family contacts, especially in highly endemic regions. Thus, the present study aimed to analyze the serum levels of MBL and FCN3 in leprosy patients and their healthy family contacts in a hyperendemic region in northeastern Brazil.

## 2. Materials and Methods

### 2.1. Study Design and Population

This was a cross-sectional study carried out with patients with leprosy and their family contacts residing in Imperatriz, Maranhão, northeastern Brazil, from July 2018 to April 2019. This city covers 1368.98 sq km and has approximately 252,320 inhabitants. In addition to presenting high levels of leprosy endemicity in recent years, it is considered a priority region for the control and surveillance of the disease [5].

Participant selection was carried out randomly and according to the established eligibility criteria. Patients of any age, of both genders, with a diagnosis of leprosy, who were currently being treated or who had already been treated for the disease, and residents of the municipality of Imperatriz, Maranhão, were recruited. In addition, healthy family contacts not undergoing treatment for leprosy or other diseases were included. Patients with leprosy who had also been diagnosed with other infections were excluded.

### 2.2. Data Collection

Data collection was performed according to patient availability while they were in outpatient care at the Municipal Health Dermatology Center and in the Primary Healthcare Units. When necessary, data collection was carried out at home during an appointment scheduled by the health and research team in advance.

The clinical characteristics of the disease were determined based on the patients’ medical records. There are four clinical forms of leprosy according to the World Health Organization [21]: indeterminate, tuberculoid, borderline, and lepromatous. This classification is used in the existing Brazilian healthcare system. The degree of physical disability was classified as Grade 0, Grade 1, Grade 2, and not assessed. Leprosy reactions were characterized as type 1 or type 2.

The FCN3 and MBL serum levels in the lectin pathway were measured from blood samples that were collected from the patients and their family contacts, with vacuum-driven devices being used for adults and butterfly collection sets being used for children or elderly subjects. Blood samples were stored in tubes with EDTA (5 mL) and 4 mL separator gel tubes. The samples that were collected in tubes without anticoagulant were centrifuged at 3000 rpm for 6 min, and the supernatant was removed and aliquoted in 1.5 mL conical tubes, which were stored at −70 °C until use.

### 2.3. MBL Dosage

The serum MBL levels were measured using an immunofluorometric assay (ELISA), adapted from Thiel, 2007. In summary, microtitration wells (Maxisorb, Nunc, Kamstrup, Skanderborg, Denmark) were coated with 10 µg of mannan (S-7504 Sigma Aldrich, St. Louis, MO, USA) diluted in a buffer coat. The residual protein binding sites were blocked with BSA (A-3294—Sigma Aldrich). SER 101 and SER 102 MBL standard serum (antibodyShop, concentrations 0.4–1.0–2.6–6.4–16–40–100 ng/mL) were used in the curve and were diluted in a binding buffer. After this step, all of the samples were diluted in the binding buffer and the controls were added to MaxiSorp polystyrene. After completing the washing and incubation procedures, biotinylated anti-MBL antibody (hyb 131-1B—antibodyShop) was added to all of wells and incubated for 2 h at 37 °C. This step was followed by streptavidin (S5742—Sigma Aldrich), TMB (3,3′,5,5′-Tetramethylbenzidine—Sigma Aldrich) with a specific incubation time, and, finally, a stop solution (1.8 M–HCL).

### 2.4. Ficolin-3 Levels (FCN3)

A commercial ELISA kit (Wuhan Fine Biotech^®^, Wuhan, China, code EH0137) was used to measure the FCN3 levels. Most publications have used Hycult^®^ kits, but we used the Fine^®^ test kit, achieving concentration curve values ranging from 0.156 to 10 ng/mL. The reproducibility and sensitivity of the kit in our laboratory were both 100%. Our values are based on the reference values from the kit.

In summary, samples and standards, all in duplicate, were added to the plate (100 μL/each well). After the plates were washed and incubated, we added 100 μL of biotinylated antibody to each plate followed by 100 μL of streptavidin –HRP and 100 μL of chromogen for incubation. Following the manufacturer’s guidelines, we added a stop solution and put the plate through an ELISA reader after 5 min at 450 nm (Biotrack II Plate Reader, Amersham Biosciences, Amersham, UK).

### 2.5. Statistical Analysis

Parametric and nonparametric tests were used to analyze the serum levels of ficolin-3 (FCN3) and MBL and the sociodemographic and clinical variables among the leprosy patients and their family contacts. The Shapiro–Wilk and Levene tests verified the normality and homogeneity of variance. Qualitative variables were assessed using the Chi-square test, and the odds ratio was calculated to measure the strength of the association. A confidence interval of 95% was established, and *p* < 0.05 was considered statistically significant. The statistical analysis was performed using the Statistical Package for the Social Sciences version 20, and GraphPad Prism 7.0 was used to analyze the clinical data and serum levels.

### 2.6. Ethical Considerations

This study was approved by an Ethics Committee for Research with Human Beings in Brazil, according to the report No. 2776066. In addition, after explaining the importance of the study, all of the patients and their family contacts signed an informed consent form, and if the patient was a child, a consent form was obtained from the child’s guardians. Participants’ confidentiality was ensured.

## 3. Results

A total of 169 subjects were analyzed: 90 were patients who had been diagnosed with leprosy and 79 were healthy family contacts. A total of 54.4% of the patients were male, 82.2% were Afro-Brazilian, and 61.2% were over 15 years of age. The patients ranged between 4 and 75 years of age, with a mean age of 29.6 (standard deviation: 20.7). Among the healthy family contacts, 64.6% were female, 74.7% were Afro-Brazilian, and 75.9% were over 15 years of age. The ages ranged between 7 and 87 years old, with a mean age of 35.3 (standard deviation: 18.7) (Table 1).

Regarding the levels of the serum proteins, it was observed that 92.2% of the patients had FCN3 concentrations above 26 µg/mL and 67.7% had MBL levels above 1000 ng/mL. Among the healthy family contacts, 94.9% had FCN3 concentrations above 26 µg/mL, and 59.4% had MBL levels above 1000 ng/mL.

Leprosy patients were more likely to be male (OR = 2.17; *p* = 0.01) and younger than fifteen years of age (OR = 2.01; *p* = 0.03).

When analyzing the serum concentrations of FCN3 and MBL, no association was observed between the protein concentrations and gender or ethnicity or whether the sample was from a patient or contact. High MBL levels (mean = 3482.6 ng/mL) were associated with an age of under fifteen years old (*p* = 0.02), as shown in Table 2.

Higher FCN3 levels were identified in patients with indeterminate leprosy (41.9 µg/mL) compared to those with the lepromatous form (*p* = 0.033). Physical disability grade 0 was also more frequently observed in patients with higher FCN3 levels (38.1 µg/mL) than those with disabilities (*p* = 0.031). Higher FCN3 levels were also observed in the group without leprosy reactions (37.4 µg/mL) compared to patients with type 1 and 2 reactions (33.7 µg/mL and 36.1 µg/mL), as shown in Table 3.

## 4. Discussion

Leprosy is a chronic inflammatory disease that persists as a public health problem in many developing countries, leading to a high rate of physical disability and social stigma as well as a high chance of affecting family and intra-household contacts [22]. The predisposition of an infected person to different immune responses can result in different clinical manifestations and can favor susceptibility or resistance, making it difficult to understand and control the disease [6,7].

In the present study, it was observed that the patients who were diagnosed with leprosy were more likely to be male and children. The above finding agrees with other studies reporting a higher occurrence of leprosy in men in most regions of the world as well as in Brazil [23,24]. The higher frequency of leprosy in males may be related to biological aspects, such as the role of testosterone in creating a favorable environment for the growth of *M. leprae*, resulting in a greater disease burden and greater clinical severity in male patients [25,26].

The leprosy cases that were investigated in the present study were more likely to occur in patients younger than 15 years old. When leprosy is detected in childhood, it indicates that family contacts have not been treated, were treated late, or have not been identified and indicates the active and recent transmission of leprosy in the community [27,28].

Studies suggest that susceptibility to leprosy and some clinical manifestations are influenced by the genetic factors of the host and indicate an immunoregulatory role of the pattern recognition molecules (PRMs) of the lectin pathway in leprosy susceptibility and clinical expression [11,12,13,14,15,16,17,18,19]. Lectin pathway components appear to be good candidates for biomarkers to determine the host’s immune response against *M. leprae*.

A study showed that polymorphisms in the FCN3 gene cooperate to increase the concentration of ficolin-3 and may contribute to leprosy susceptibility by favoring the spread of *M. leprae* [19]. On the other hand, low levels of MBL and a deficiency in complement activation confer resistance to the development of leprosy and borderline clinical forms of leprosy [16].

In the present study, it was observed that the serum levels of MBL were higher in children under 15 years of age and that they were not associated with the clinical manifestation or severity of leprosy. The lack of a genetic characterization of MBL was a limiting factor of the study. However, the values that were obtained were compared with those obtained from members of the same family.

A recent study investigated MBL2 gene polymorphism and serum MBL levels in patients with leprosy, demonstrating that age can influence the MBL2 phenotype. Patients under 40 years of age had decreased MBL levels compared to those of over 40 years, but there is no evidence of MBL2 polymorphism being associated with susceptibility to leprosy or its clinical forms [29].

MBL and FCN-3 levels are higher in children than in adults [30]. The expression of both molecules appears to be epigenetically regulated during development, and differences compared to older patients may be a consequence of this regulation rather than the disease.

The serum values of FCN3 and MBL that were found in this study were not different between patients with leprosy and the healthy relatives, suggesting that the serum levels of these lectins did not contribute to increased susceptibility. However, patients with mild and transient clinical manifestations of leprosy had high FCN3 serum levels, with higher prevalence being observed in the indeterminate form, as well as an absence of physical disabilities and leprosy reactions.

The role of ficolins in the interaction with the surfaces of *M. leprae* and host cells has not yet been established. One study showed that the functional haplotypes that produce serum FCN2 eliminate *M. leprae* via mononuclear phagocytes and protect subjects against leprosy [18]. Another study investigating polymorphisms in the FCN1 gene showed a negative association of a haplotype with lepromatous leprosy compared to less severe forms of the disease. Thus, the production of ficolins can play a protective role in severe forms of the disease and are important biological markers for understanding *M. leprae* infection [17].

A study that investigated the polymorphism of the FCN3 gene and FCN3 serum production showed that the polymorphisms were not associated with leprosy. However, high serum concentrations of ficolin-3 were associated with the lepromatous form of the disease, which is probably caused by polymorphisms in the FCN3 gene, which may contribute to leprosy susceptibility by favoring the infection [19].

During the development of this research, several limitations were encountered, such as the fact that MBL and FCN3 genes have not been genetically characterized and the study’s cross-sectional nature. Cross-sectional studies collect the data of the exposure variable and the outcome at the same time, in order to describe characteristics of the sample or to study associations. Using these studies, associations of interest for research can be established, but no causal relationships should be inferred, as there is no follow-up. Despite the limitations, the results of the present study provide initial evidence promoting the measurement of the serum proteins in the complement system in patients and in family contacts in regions that are hyperendemic for leprosy and offers subsidies for the development of future research to genetically characterized the MBL and FCN3 genes and a better understanding of the immunopathogenesis of *M. leprae* infection.

## 5. Conclusions

The present study showed that serum MBL levels were higher in children under 15 years of age than they were in adults. Higher serum FCN3 levels were associated with the early and transient clinical forms of leprosy and lower expression in severe forms of the disease.

## Figures and Tables

**Table 1:** Distribution of sociodemographic characteristics and serum concentrations of FCN3 and MBL in leprosy patients and healthy family contacts.

	Patients *n* = 90 *n* (%)	Family Contacts *n* = 79 *n* (%)	*p*-Value	OR	95% CI
**Gender**					
Male	49 (54.4)	28 (35.4)	0.01 *	2.17	1.17–4.04
Female	41 (45.6)	51 (64.6)			
**Age**					
<15 years	35 (38.8)	19 (24.1)	0.03 *	2.01	1.03–3.91
>15 years	55 (61.2)	60 (75.9)			
**Ethnicity**					
Afro-Brazilian	74 (82.2)	59 (74.6)	0.23	1.56	0.79–3.29
Euro-Brazilian	16 (17.8)	20 (25.4)			
**FCN3 (µg/mL)**					
>26 µg/mL	83 (92.2)	75 (94.9)	0.47	0.63	0.17–2.24
<26 µg/mL	7 (7.8)	4 (5.1)			
**MBL** **(ng/mL)**					
>1000 ng/mL	61 (67.7)	47 (59.4)	0.26	1.43	0.76–2.69
<1000 ng/mL	29 (32.3)	32 (40.6)			

* *p* < 0.05; OR = odds ratio; 95% CI = 95% confidence interval.

**Table 2:** Associations between FCN3 and MBL concentrations and sociodemographic characteristics in leprosy patients and healthy contacts (*n* = 169).

Variables	No	FCN3 (µg/mL)		MBL (ng/mL)	
		Mean (SD)	*p*-Value	Mean (SD)	*p*-Value
**Gender**					
Male	77	36.8 (66.2)	0.34	3151.7 (2324.4)	0.21
Female	92	36.1 (73.9)		2688.8 (2354.1)	
**Age**					
<15 years	54	37.4 (71.3)	0.25	3482.6 (2468.8)	0.02 **
>15 years	115	35.9 (69.8)		2626.0 (2243.7)	
**Ethnicity**					
Afro-Brazilian	133	36.2 (70.3)	0.44	2828.9 (2367.0)	0.43
Euro-Brazilian	36	37.2 (71.1)		3161.5 (2275.2)	
**Cases**					
Patients	90	36.3 (70.9)	0.76	3035.9 (2264.1)	0.29
Healthy contacts	79	36.5 (70.2)		2744.6 (2439.3)	

** Mann–Whitney test *p* < 0.05; SD: standard deviation.

**Table 3:** Association between FCN3 and MBL serum concentrations and clinical factors in leprosy patients.

	No	FCN3 (µg/mL)		MBL (ng/mL)	
		Mean (SD)	*p*-Value	Mean (SD)	*p*-Value
**Clinical form**					
Indeterminate		41.9 (49.9)	0.03 *	3324.9 (2087.0)	0.77 ***
Tuberculoid		34.5 (65.6)		2616.4 (2523.9)	
Borderline		36.5 (75.6)		3002.9 (2308.8)	
Lepromatous		34.3 (60.0)		3260.8 (2176.0)	
**Operational classification**					
Multibacillary		35.9 (71.2)	0.362 **	3075.9 (2239.1)	0.70 ****
Paucibacillary		37.5 (70.5)		2919.3 (2382.8)	
**Hansenic reaction**					
None		37.4 (69.2)	0.10 *	2794.3 (2100.3)	0.51 ***
Type 1		33.7 (73.6)		3561.2 (2696.9)	
Type2		36.1 (62.2)		3210.9 (1972.6)	
**Treatment status**					
In treatment		37.2 (71.1)	0.21 **	2930.4 (2346.8)	0.58 ****
Previously treated		35.3 (69.6)		3156.4 (2187.6)	
**Degree of disability**					
Grade 0		38.1 (66.9)	0.03 *	3272.0 (2155.8)	0.25 ***
Grade I		34.3 (83.3)		2304.6 (2479.1)	
Grade II		32.7 (68.9)		3353.6 (2602.5)	
Not assessed		35.1 (59.8)		2189.0 (1945.8)	

* Analysis of variance (ANOVA) with Duncan’s post hoc test. ** Student’s *t*-test. *** Kruskal–Wallis test (with multiple comparisons using the Nemenyi test). **** Mann–Whitney test.

## Data Availability

The data supporting this study can be accessed by readers freely through the availability of the same by the authors, as long as the participant’s personal data is preserved.
